# Ambient air pollution and emergency department visits among children and adults in Casablanca, Morocco

**DOI:** 10.3934/publichealth.2021022

**Published:** 2021-03-22

**Authors:** Chakib Nejjari, Abdelghafour Marfak, Ahmed Rguig, Abderrahmane Maaroufi, Ihssane El Marouani, Abderrafii El Haloui, Bouchra El Johra, Rachid Ouahabi, Rachid Moulki, Amina Idrissi Azami, Youness El Achhab

**Affiliations:** 1 Laboratory of Epidemiology, Clinical Research and Community Health, Faculty of Medicine and Pharmacy of Fez, Morocco; 2 Mohammed VI University for Health Sciences, Casablanca, Morocco; 3 National School of Public Health, Rabat, Morocco; 4 Laboratory of Health Sciences and Technology, Higher Institute of Health Sciences, Hassan 1^st^ University of Settat, Morocco; 5 Ministry of Health, Rabat, Morocco; 6 Mohammed VI Foundation for Environmental Protection, Casablanca, Morocco; 7 General Directorate of Meteorology of Morocco in Casablanca, Morocco; 8 Regional Health Directorate of Casablanca-Settat, Casablanca, Morocco; 9 Regional Observatory of Epidemiology of Casablanca, Morocco; 10 Regional Center for Careers Education and Training, Fez-Meknes, Morocco

**Keywords:** air pollution, conditional poisson model, environmental health, respiratory diseases, emergency department visits

## Abstract

This study presents the relationships between ambient air pollutants and morbidity and emergency department visits among children and adults performed in Great Casablanca, the most populated and economic region in Morocco. This research was analyzed using conditional Poisson model for the period 2011–2013. In the period of study, the daily average concentrations of SO_2_, NO_2_, O_3_ and PM_10_ in Casablanca were 209.4 µg/m^3^, 61 µg/m^3^, 113.2 µg/m^3^ and 75.1 µg/m^3^, respectively. In children less than 5 years old, risk of asthma could be increased until 12% per 10 µg/m^3^ increase in NO_2_, PM10, SO_2_ and O_3_. In children over 5 years and adults, an increase of 10 µg/m^3^ air pollutant can cause an increase until 3% and 4% in respiratory consultations and acute respiratory infection, respectively. Similarly, impact on emergency department visits due to respiratory and cardiac illness was established. Our results suggest a not negligible impact on morbidity of outdoor air pollution by NO_2_, SO_2_, O_3_, and PM_10_.

## 1. Introduction

Air pollution is now considered to be a significant public health issue, responsible for an increasingly range of health effects that are well recognized from epidemiological studies conducted in many regions of the world [1]–[3]. The latest estimate from the World Health Organization (WHO) reported that in 2012, approximately 3.7 million deaths were attributable to ambient air pollution and about 88% of these deaths occur in low- and middle-income countries, which represent 82% of the world population [4]. According to recent studies performed in the metropolises, a statistically significant association was observed between particulate matter, gaseous pollutants and mortality and department admissions for respiratory and cardiovascular diseases [5]–[8]. Furthermore, several studies confirmed that people from around the world are still exposed to concentrations exceeding the WHO recommendations attracting a great deal of attention from the government and the public [9]–[11].

The 2005 “WHO Air quality guidelines” offer global guidance on thresholds and limits for key air pollutants: particulate matter (PM), ozone (O_3_), nitrogen dioxide (NO_2_) and sulfur dioxide (SO_2_), which pose health risks in all WHO regions [12]. PM consists of a complex mixture of solid and liquid particles of organic and inorganic substances suspended in the air. The most health-damaging particles are those with a diameter of 10 microns or less (PM_10_), which can penetrate and lodge deep inside the lungs [10]. Ozone is one of the major constituents of photochemical smog and it has been associated with increases in mortality and hospital admissions due to respiratory and cardiovascular disease [13]–[15]. Largest epidemiologic studies of air pollution suggests that short-term exposure to NO_2_ [16] and SO_2_ [17] is associated with adverse health effects and increased mortality risk. In a recent study in Beijing, the authors concluded that for lag 2, a 10 µg/m^3^ increase in concentration of PM_10_, SO_2_ and NO_2_ were associated with 1.7%, 1.3%, and 2.6% increases respectively in respiratory disease emergency admissions [18]. Also, it has been shown that PM_10_ was associated to antibiotics use [19].

More recently, it has been demonstrated that air pollution is also associated with the spread of COVID-19 [20],[21]. Authors found that an increase in NO_2_, and O_3_ result in increase in the daily counts of confirmed cases. Conversely, an increase of SO_2_ result in decrease in COVID-19 confirmed cases. The relation among atmospheric stability based on wind speed, air pollution and the spread of COVID-19 has been well established [21]–[26]. Cities with low wind speed and frequently high levels of air pollution had higher numbers of COVID-19 cases.

Due to its high population density, intense industrial activities and large traffic volume, the Great Casablanca (GC) is suffering from intense air pollution during the last two decades [27]–[29]. However, there are rare studies on the potential health effects of air pollution in Morocco and other African countries. This first study aimed to analyze the short-term effects of PM_10_, SO_2_, NO_2_ and O_3_ on the exposed population in the GC, notably the association between air pollution and respiratory morbidity and related-treatment.

## 2. Materials and methods

### 2.1. Study area

**Figure 1.**
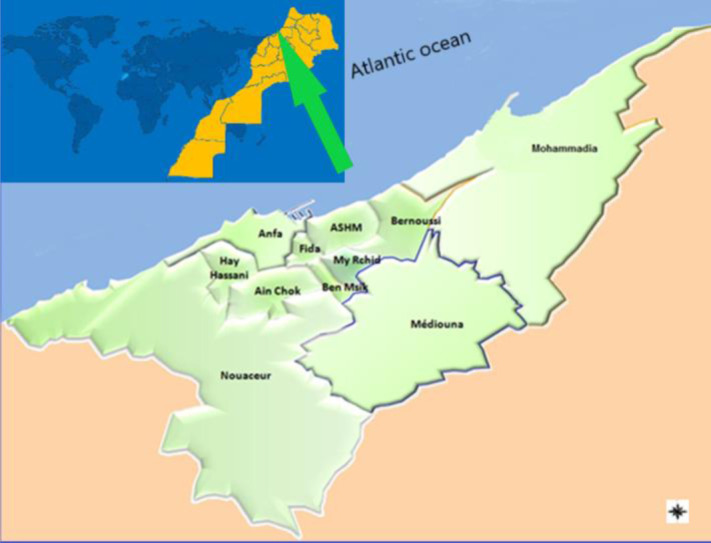
Map of Great Casablanca and their world location.

Casablanca is the largest city of Morocco, located in the central-western part of the country on the Atlantic Ocean (33°32′N7°35′W) with an urban population of 3.71 million inhabitants and an area of 220 km^2^. The Great Casablanca (GC) consisted of two prefectures and two provinces: Prefecture of Casablanca; Prefecture of Mohammedia; Nouaceur Province and Mediouna Province (Figure 1). The Greater Casablanca region is considered the locomotive of the development of the Moroccan economy. Casablanca is the industrial center of Morocco with more than half of the country's factories [28]. The study on atmospheric emission inventory, for the year 1992 in the GC, showed very large emissions of pollutants mainly due to the presence of a refinery and several power plants [27].

### 2.2. Air pollutant and meteorological data collection

Daily concentrations of O_3_, NO_2_, SO_2_ and PM_10_ (µg/m^3^) and daily relative humidity (%) and temperature (°C) during the study period were obtained from the Meteorological Administration. The four pollutants were measured by seven automated environmental monitoring stations installed in 7 geographical zones in Casablanca. These stations measure each 15 minutes air pollution levels originating from exhaust gasses and other sources of pollution. SO_2_ is measured by fluorescence ultraviolet according to the EN 14212 method, NO_2_ by chemiluminescence analyzer according to the EN 14211 method, O_3_ by ultraviolet absorption according to the EN 14625 method and PM_10_ by gravimetric method. For each day, we calculated daily concentration for each pollutant as the average of the 96x7 measurements (1 measurement each 15 min during 24 hours by each station). The relative humidity was measured using a psychrometer installed 2 meters above ground level in a louvered, double-roofed shelter. All data were collected from November 1, 2011 to October 31, 2013.

### 2.3. Morbidity data collection

The study sample represents all residents from the GC. Data on morbidity (hospitalization and consultation) of residents in the corresponding areas were extracted from different structures: university hospital center, 9 hospitals, 35 primary care centers, 9 respiratory diseases centers, 11 communal hygiene offices. Hospitalization data refer to public and private hospitals. Consultations data of children (under 5 years) and of adults and youths were extracted from public and private hospitals, respiratory diseases centers and primary care centers. At the end of each day, the physicians filled out a form designed for this study and sent it to the data collection coordinator. This form included the socio-demographic characteristics and the health problem constituting the object of the consultation at the health facility. All morbidity data were collected from November 1, 2011 to October 31, 2013. The morbidity variables were upper acute respiratory infection (UARI, ICD-10: J06.9), lower acute respiratory infection (LARI, ICD-10: J22), pneumonia (PN, ICD-10: J18.9), severe pneumonia (SPN), asthma (ICD-10: J45), sore throat (ST, ICD-10: R07.0), earaches (ICD-10: H92.09), very serious diseases (VSD), respiratory consultations (RC), acute respiratory disease (ARD), conjunctivitis (ICD-10: H10.9) and chronic obstructive pulmonary disease (COPD, ICD-10: J44.9). We also collected the number of antibiotic and bronchodilator prescriptions. We divided the study data into two sets of data depending on the nature of the disease. Thus UARI, LARI, PN, SPN, asthma, ST, earaches, and VSD have been studied in children under 5 years. RC, ARD, conjunctivitis and COPD have been studied in children over 5 years and adults. Only asthma, prescriptions of antibiotics and bronchodilators have been studied in all age categories.

### 2.4. Morbidity and air pollutants statistical description

Concentrations of air pollutants and morbidity data were summarized by daily mean, median, maximum, minimum, standard deviation and inter quartile interval. In addition, for each pollutant we calculated the exceeding standard ratio (ESR). This ratio is defined as the number of days when the daily concentration of the pollutant is above the standard limit value to the total number of days (731 days) of the period study. Based on Moroccan National Ambient Air Quality Standards the standards limit values are 125 µg/m^3^ for SO_2_, 50 µg/m^3^ for NO_2_, 110 µg/m^3^ for O_3_ and 50 µg/m^3^ for PM_10_.

### 2.5. Statistical modelling

To analyze the association between daily concentrations of air pollutants and morbidity data the generalized additive models (GAMs) with Poisson regression have been used [30]–[32]. In this model the log mean morbidity was modeled as a sum of terms representing the covariates. Confounding factors, meteorological variables and time, were modeled in a flexible manner as nonlinear smooth functions of time and air pollutants were modeled in a linear manner. Recently, Armstrong and colleagues proposed the conditional Poisson model in which primary confounder control is by stratifying time by month and day-of-week, a typical case crossover approach. The conditional Poisson model is a multinomial model [33]:

$$Y_{i,s}|Y_{.,s}\sim Multinomial\left(\left\{\pi_{i}\right\}\right),\;\pi_{i}=\frac{exp\left\{\beta^{T}x_{i}\right\}}{\sum_{j\in s}exp\left\{\beta^{T}x_{j}\right\}}$$ (1)

where the confounder control time strata (month and day-of-week) are denoted *s* = 1, ..., *S*. *Y_i_* is the count of morbidity outcome at day “*i*”. ***x_i_*** is the air pollutants, temperature and relative humidity vector. *β* is the vector of parameter being estimated by conditioning on the sum of events *Y*_.,*s*_ = Σ_*i*_*Y*_*i*,*s*_ in each stratum (more details in [33]). A plausible model was chosen using as a guide Akaike's information criterion (AIC) which is an estimate of expected prediction error [34].

For each model we tested the association of the morbidity variables and the concentrations of the air pollutants for the same day (lag0) up to 4 lag days (lag4). From these four lag days models the model that yielded the smallest *p-value* was chosen. All calculations were performed with the R software (R version 4.0.3 (2020-10-10)) using the “gnm” package.

## 3. Results

### 3.1. Descriptive statistics of air pollutants

**Table 1.** Summary of daily concentrations (µg/m^3^) of SO_2_, NO_2_, O_3_ and PM_10_ in Casablanca from 11/01/2011 to 10/31/2013.

	SO_2_	NO_2_	O_3_	PM_10_
Mean	209.4	61	113.2	75.1
Standard deviation	218.4	38.7	147.6	52.4
Interquartile range	298.7	46	80	33
Minimum	5.8	0	14	7
Lower quartile	33.3	32	32	50
Median	126	50	67	64.7
Uper quartile	332	78	112	83
Maximum	904	251	812	673
ESR* (%)	23.8	2.7	15.3	15.1

Note: *ESR: Exceeding Standard Ratio.

**Figure 2.**
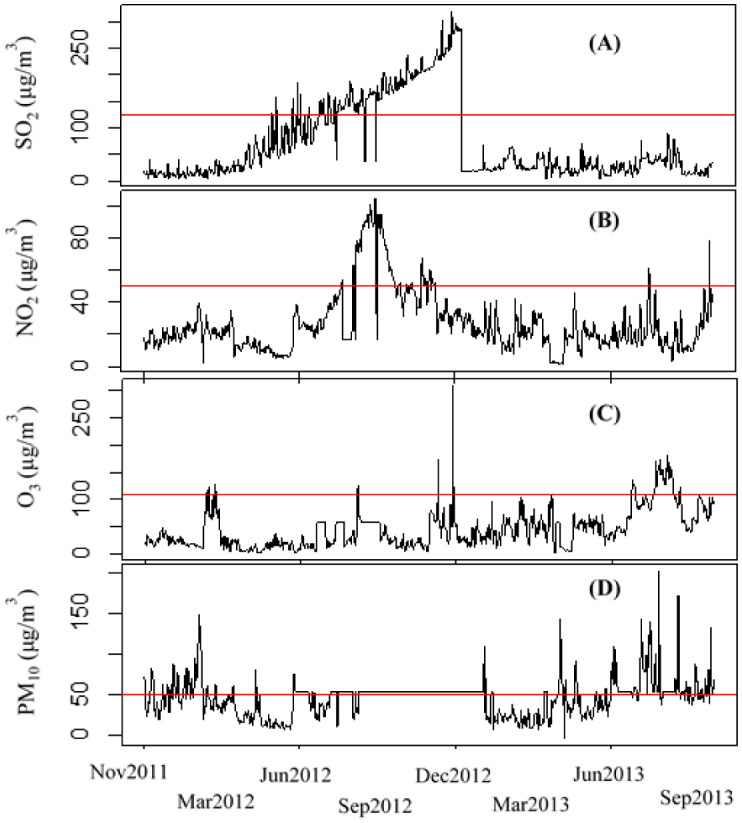
Mean daily concentration distributions of SO_2_ (A), NO_2_ (B), O_3_ (C), and PM_10_ (D) in Great Casablanca during 11/01/2011 to 10/31/2013. The horizontal lines represent the standard daily average limits of SO_2_ (125 µg/m^3^), NO_2_ (50 µg/m^3^), O_3_ (110 µg/m^3^) and PM_10_ (50 µg/m^3^) in Moroccan National Ambient Air Quality Standards.

Descriptive analysis showed that the mean daily concentrations of SO_2_, NO_2_, O_3_ and PM_10_ in GC were 209.4 µg/m^3^, 61 µg/m^3^, 113.2 µg/m^3^ and 75.1 µg/m^3^, respectively (Table 1). According to the interquartile range and the standard deviation values, we observed that SO_2_ had the highly daily variation in comparison to the three other pollutants. In contrast, NO_2_ had low fluctuations during the period study. The daily variations of the four air pollutants are shown in Figure 2. Level concentrations of SO_2_ increased in 2012 and decreased in 2013. NO_2_ observed fluctuations during the period study. Although NO_2_ had fluctuations during the period study, it had the low ESR (2.7%). The highest ESR value was obtained for SO_2_ (23.8%) followed by O_3_ (15.3%) and PM_10_ (15.1%). We concluded that the SO_2_ pollution is more serious than the other pollutants.

### 3.2. Descriptive statistics of morbidity

In children under 5 years, a total of 218,181 consultations were recorded (Table 2). The UARI accounted for 19.4% followed by sore throat with 14.0% and LARI (11.4%). The daily average of the three variables was 68 (SD = 47.8), 49 (SD = 31.2) and 40 (SD = 30.6), respectively (Table 3). Pneumonia come in fourth position with 8.0% followed by earaches (5.9%) with daily averages of 28 (SD = 21.0) and 21 (SD = 14.5), respectively. For asthma we recorded 2 cases on average per day. The rate of VSD and SPN did not exceed 1%. For the 8 variables studied, we observed that the morbidity rate is higher in children between 24 months and 59 months (Table 2). We observed that antibiotic prescriptions correspond to a ratio of 0.3; i.e; an antibiotic prescription for one per 3 consultations. Bronchodilators are less prescribed than antibiotics (Table 2).

**Table 2.** Percentage of consultations adjusted on age category for children under 5 years in Casablanca during 11/01/2011 to 10/31/2013.

	TC	UARI	ST	LARI	PN	Earaches	ASTHMA	SVD	SPN	Antibiotic Prescriptions	Bronchodilator Prescriptions
0–11 months	68,418 (31%)	8,653 (20%)	4,269 (14%)	7,686 (31%)	5,693 (32%)	3,528 (28%)	313 (22%)	203 (27%)	247 (38%)	15,646 (23%)	449 (26%)
12–23 months	62,988 (29%)	13,537 (32%)	9,475 (31%)	7,716 (31%)	4,985 (29%)	4,024 (31%)	356 (26%)	247 (33%)	198 (31%)	20,468 (31%)	456 (27%)
24–59 months	86,775 (40%)	20,079 (48%)	16,889 (55%)	9,535 (38%)	6,807 (39%)	5,257 (41%)	725 (52%)	306 (40%)	204 (31%)	30,701 (46%)	798 (47%)
Total	218,181	42,269	30,633	24,937	17,485	12,809	1394	756	649	66,815	1,703
% of morbidity		19.4%	14.0%	11.4%	8.0%	5.9%	0.6%	0.4%	0.3%	

Note: TC: Total Consultations, UARI: Upper Acute Respiratory Infection, ST: Sore Throat, LARI: Lower Acute Respiratory Disease, PN: Pneumonia, SVD: Serious Disease, SPN: Serious Pneumonia.

**Table 3.** Summary of daily clinic consultations for children under 5 years in Casablanca during 11/01/2011 to 10/31/2013.

	TC	UARI	ST	LARI	PN	Earaches	Asthma	SVD	SPN	Antibiotic Prescriptions	Bronchodilator Prescriptions
Mean	351	68	49	40	28	21	2	1	1	108	3
Standard deviation	188.3	47.8	31.2	30.6	21.0	14.5	2.9	14.3	1.9	65.6	3.7
Interquartile range	221	56	37	37	29	20	3	0	1	89	4
Minimum	0	0	0	0	0	0	0	0	0	0	0
Lower quartile	255	35	29	18	12	9	0	0	0	60	0
Median	387	65	49	35	25	20	1	0	0	113	1
Upper quartile	476	91	66	55	41	29	3	0	1	149	4
Maximum	1022	362	184	176	107	82	20	351	16	333	24

Note: TC: Total Consultations, UARI: Upper Acute Respiratory Infection, ST: Sore Throat, LARI: Lower Acute Respiratory Disease, PN: Pneumonia, SVD: Serious Disease, SPN: Serious Pneumonia.

**Table 4.** Percentage of consultations adjusted on age category for children above 5 years and adults in Casablanca during 11/01/2011 to 10/31/2013.

	TC	RC	ARI	Asthma	Conjunctivitis	COPD	Antibiotic Prescriptions	Bronchodilator Prescriptions
5–14 years	134,325 (20%)	27,648 (28%)	19,637 (30%)	1,598 (14%)	2,957 (36%)	114 (14%)	30,568 (27%)	1,439(15%)
15–50 years	348,043 (50%)	44,518 (45%)	29,649 (45%)	6,344 (57%)	3,603 (43%)	342 (43%)	52,179 (46%)	5,212 (56%)
>50 years	205,658 (30%)	26,161 (27%)	16,694 (25%)	3,234 (29%)	1,753 (21%)	346 (43%)	30,457 (27%)	2,709 (29%)
Total	688,026	98,327	65,980	11,176	8,313	802	113,204	9,360
% Of morbidity		14.0%	10.0%	2.0%	1.0%	0.12%	

Note: TC: Total Consultations, RC: Respiratory Consultations, ARI: Acute Respiratory Infection, COPD: Chronic Obstruction Pneumonia Disease.

**Table 5.** Summary of daily clinic consultations for children above 5 years and adults in Casablanca during 11/01/2011 to 10/31/2013.

	TC	RC	ARI	Asthma	Conjunctivitis	COPD	Antibiotic Prescriptions	Bronchodilator Prescriptions
Mean	1117	160	107	18	13	1	184	15
Standard deviation	563.3	109.5	86.6	13.3	9.1	1.9	113.1	11.6
Interquartile range	668	157.3	116	16	12	2	161.3	14
Minimum	0	0	0	0	0	0	0	0
Lower quartile	834.3	78.8	41	9	7	0	99.8	7
Median	1303.5	149.5	90.5	18	13	1	198	14
Upper quartile	1502.3	236	157	25	19	2	261	21
Maximum	2261	537	545	128	54	16	469	67

Note: TC: Total Consultations, RC: Respiratory Consultations, ARI: Acute Respiratory Infection, COPD: Chronic Obstruction Pneumonia Disease.

In children over 5 years and adults, a total of 688,026 consultations were recorded for the two years of the study (Table 4 and Table 5). 14% of all consultations were for respiratory causes (98,327) and 10% for acute respiratory disease with daily averages of 160 (SD = 109.5) and 107 (SD = 86.6), respectively. The average daily asthma was 18 (SD = 13.3) and the conjunctivitis 13 (SD = 9.1) with a total number of 11,176 and 8,313, respectively. The highest morbidity rate was observed for the age group between 15 and 50 years. Regarding antibiotic prescriptions, we found that an antibiotic is prescribed for one consultation on 6. As for the sub-group of the children under 5 years, bronchodilators are prescribed fewer antibiotics.

### 3.3. Association between air pollution and morbidity: results of conditional Poisson models

**Figure 3.**
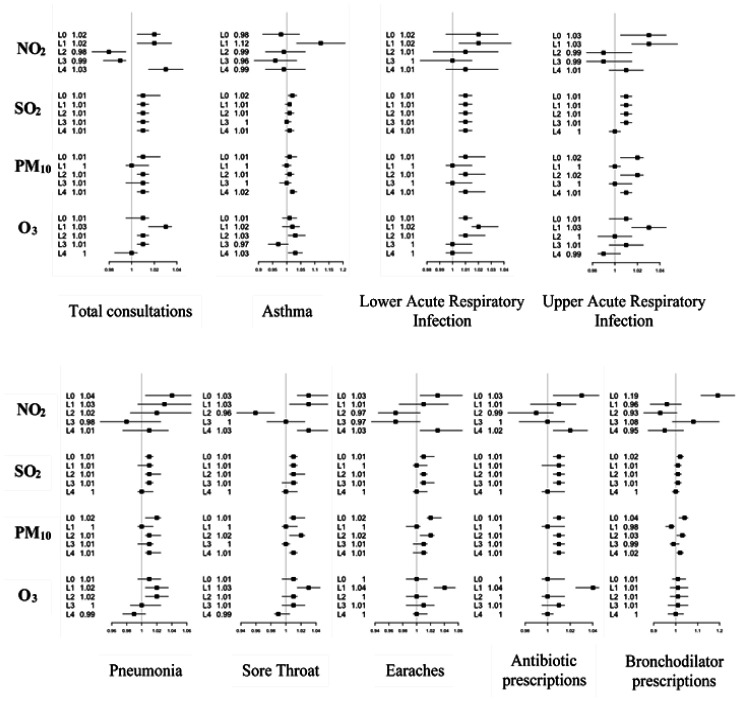
Relative risks (RRs with 95% CI) of upper acute respiratory infection (UARI), lower acute respiratory infection (LARI), pneumonia (PN), severe pneumonia (SPN), asthma, sore throat (ST), earaches and prescription of antibiotics and bronchodilators in association with per 10 µg/m^3^ increase in O_3_, NO_2_, PM_10_ and SO_2_ for children under 5 years in the population of Great Casablanca at different lag days (L0: lag0, L1: lag1, L2: lag2, L3: lag3 and L4: lag4).

**Figure 4.**
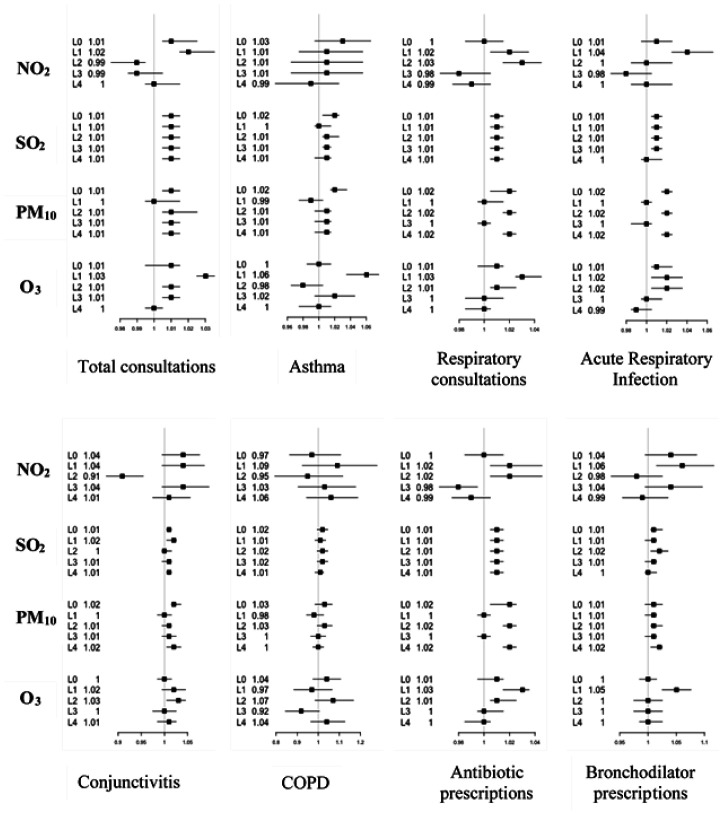
Relative risks (RRs with 95% CI) of acute respiratory disease (ARD), respiratory consultations (RC), COPD, asthma, conjunctivitis and prescription of antibiotics and bronchodilators in association with per 10 µg/m^3^ increase in O_3_, NO_2_, PM_10_ and SO_2_ for children above 5 years and adults in the Great Casablanca population at different lag days (L0: lag0, L1: lag1, L2: lag2, L3: lag3 and L4: lag4).

Five lag days labeled lag0 (the current day), lag1 (one day after), lag2 (two days after), lag3 (three days after) and lag4 (four days after) were studied. The relative risks obtained for each pollutant and each day lag were shown in Figure 3 for children under 5 years and in Figure 4 for children over 5 years and adults.

In children under 5 years, we observed a significant association between the 9 studied morbidity variables and exposition to NO_2_ for the current day (except asthma) and the next day (lag1, except bronchodilator prescriptions). This association decreased for lag2 and lag3. An increase of 10 µg/m^3^ in NO_2_ induced an increase of 12% of asthma cases; 3% of UARIs, pneumonia and sore throat; and 2% of LARIs in the second day after exposition. In the current day of exposition to NO_2_, bronchodilator and antibiotic prescriptions were increased by 19% and 3%, respectively; and decreased for the days after. For PM_10_, no association was observed for the second day and all morbidity variables. However, for the current day an increase of 10 µg/m^3^ would lead to 2% increase of LARIs, 2% of PNs, 2% of earaches and 1% of STs. This increase was accompanied with 4% and 1% increase in bronchodilator and antibiotic prescriptions, respectively. Regarding SO_2_, for each day the exposition to the pollutant showed similar effect on morbidity in children under 5. An increase of 10 µg/m^3^ of SO_2_ was accompanied with 1% to 2% of morbidity. The pollutant O_3_ showed significant effect on morbidity of all morbidity variables, specifically in the first day after exposition (lag1) in children under 5 years. Figure 3 showed that the relative risks for morbidity were gradually weak for lag3 and lag4. Bronchodilator prescriptions were not associated to exposition to O_3_.

In children over 5 years and adults, results showed that SO_2_ was significantly associated with acute respiratory infections (RR of 1.01), conjunctivitis (RR of 1.01), COPD (RR of 1.02 for lag0; and 1.01 for lag1), and asthma (RR of 1.02 for lag1 and 1.01 for lag2 and lag3) (Figure 4). The increase of morbidity due to SO_2_ exposition was accompanied to 1–2% of antibiotics and bronchodilators prescriptions. Asthma is also associated with pollutant PM_10_. An increase of 10 µg/m^3^ of PM_10_ can cause an increase of 2% in the number of asthma cases on the day of the exhibition (lag0) and 1% for the next days (lag2 and lag3). PM_10_ was also associated with respiratory consultations (2%) and ARIs (2%). For drug prescriptions, a significant association was obtained between PM_10_ and bronchodilators for lag2 (1%); and antibiotics for lag0, lag2 and lag4 with 2% (Figure 4). The impact of NO_2_ was observed in the first day after exposition (lag1) where an increase of 10 µg/m^3^ could give an increase of 2% in the number of respiratory consultations and 4% of ARIs. Treatment prescriptions were 2% and 6% for antibiotics and bronchodilator, respectively. As for NO_2_, the effected of O_3_ was significant for day lag1, notably for asthma (6%), respiratory consultations (3%) and ARIs (2%) accompanied by 3% and 5% of antibiotic and bronchodilator prescriptions, respectively.

### 3.4. Emergency department visits

**Figure 5.**
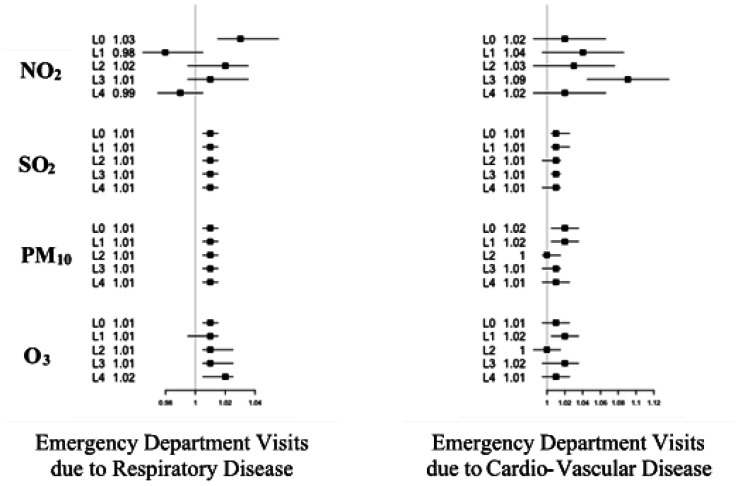
Relative risks (RRs with 95% CI) of Emergency Department Visits (EDVs) due to respiratory diseases and cardiac diseases in association with per 10 µg/m^3^ increase in O_3_, NO_2_, PM_10_ and SO_2_ at different lag days (lag0 = L1, lag1 = L2, lag2 = L3, lag3 = L4).

We also studied the relationships between ambient air pollutants (PM_10_, SO_2_, NO_2_ and O_3_) and emergency department visits (EDVs) for respiratory and cardiac diseases from 2011 to 2013 in Casablanca. Figure 5 showed forest plot for the relative risks estimated using conditional Poisson model for lag0, lag1, lag2, lag3 and lag4 modeling the association between the daily average of the pollutant level and the daily number of EDVs for respiratory diseases (RDs) and cardiac diseases (CDs).

Figure 5 shows that exposure to ozone pollutant contributed to the number of emergency department visits for respiratory causes with 1% (for lag0, lag2 and lag3) and 2% for lag4. A significant effect of O_3_ on emergency department visits due to cardio-vascular disease was only observed for lag1 (2%). For NO_2_, exposure on the current day did not affecte immediately the number of EDVs due to cardio-vascular diseases. However, an increase of 10 µg/m^3^ of NO_2_ level induced significantly an increase of these visits three days after exposition (9%). EDVs due to respiratory diseases increased by 3% in lag0 and decreased on days after. Regarding SO_2_, the relative risk for EDVs due to cardiac and respiratory diseases remained constant (1%) for all days. Regarding PM_10_, the impact on EDVs due to respiratory diseases was the same as that of SO_2_. PM_10_ could contribute significantly with 2% to the number of EDVs due to cardiac diseases for lag0 and lag1.

## 4. Discussion

To assess the effect of air pollution on the Casablanca population morbidity, we studied the association of daily concentrations of the O_3_, NO_2_, SO_2_ and PM_10_ pollutants and daily number of both consultations due to respiratory diseases and emergency department visits due to respiratory and cardiac diseases. Association between each pollutant and each morbidity variable (UARI, LARI, PN, SPN, asthma, ST, earaches, VSD, RC, ARD, conjunctivitis, COPD, respiratory disease EDVs and cardiac disease EDVs) and antibiotic and bronchodilator prescriptions was studied using a conditional Poisson. Our results showed that one could predict an increase in the number of EDVs due to cardiac diseases of 2% to 9%, of 1%, of 1% to 2% and of 1% to 2% caused by an increase of 10 µg/m^3^ in the NO_2_, SO_2_, PM_10_ and O_3_ pollutant concentrations, respectively. The number of EDVs due to respiratory diseases was also affected by an increase of the air pollutants.

Particulate matters are mainly discharged from various automobiles and harbor a more complex impact on asthmatic airways, since their deposition in the airways directly elicited airway inflammation, mucosal edema and cytotoxicity [35]. In a recent review, Jang *et al*. reported that PM_10_ exposure significantly increased hospital admissions for all conditions studied, including asthma, allergic rhinitis, and upper and lower respiratory tract diseases [36]. Regarding results we obtained for PM_10_, it was found associated with increasing emergency hospital admissions for respiratory and cardiac diseases and consultations for asthma (12% in children under 5 years and 2% in children above 5 years and adults, respectively). Similar results were obtained by Hajat et al. [37]. PM_10_ was found to increment the relative risk of the number of pneumonia consultations (RR = 1.02), COPD (RR = 1.03 for lag3) and the emergency department visits (RR = 1.01 to 1.02). A study conducted by Zanobetti et al. in 10 US cities indicated that a daily 10 µg/m^3^ increase of PM_10_ resulted in a 2.5% increase in COPD, a 1.9% increase in pneumonia, and a 1.2% increase in cardiovascular diseases [38]. A recent study showed that a 10 µg/m^3^ increase in the PM_10_ concentration is associated with adverse health effects to the human respiratory system [39].

There is evidence that NO_2_ contributes to the public health burden of air pollution. In a recent review, Mills et al. have established that there are significant associations between exposure to NO_2_ and mortality and hospital admissions for a range of respiratory and cardiovascular diseases in different age groups [16]. In our study, NO_2_ was significantly associated to lower acute respiratory infection in children under 5 years with an increase of 2% (lag2) corresponding to an increase of 10 µg/m^3^ of the air pollutant. The same result has been reported previously [40]. Also, NO_2_ induced a change of 2–4% of pneumonia consultations and 3% of sore throat. There was moderate evidence that exposure to an annual mean below 40 µg/m^3^ of NO_2_ is associated with adverse health effects in susceptible populations as four studies were conducted among children and adolescents [41]–[44]. We also found that NO_2_ had significantly effect at lag1 (one day after exposure to the pollutant) than at lag0. This result was also observed in the eastern china population study conducted by Liu et al. [45]. More recently, Pannullo et al. demonstrated association between NO_2_ and respiratory hospital admissions [46].

Sulfur dioxide is a respiratory irritant and bronchoconstrictor, and has been associated with cardiovascular abnormalities [47],[48]. Relative to our finding, in the current day of exposure to SO_2_, the pollutant immediately induced a relative risk of 1% of EDVs due to respiratory and cardiac diseases. This could be explained by the fact that inhalation of SO_2_ leads to rapid onset bronchoconstriction [49]. Yet, SO_2_ was associated to 2% to 2% of bronchodilator prescriptions in children under 5 years and in children above 5 years and adults. We found that SO_2_ caused an increase in the consultation number for asthma of 2% in children under 5 years and in children above 5 years and adults. Recently, studies revealed that SO_2_ had physiological effects on the cardiovascular system, including vasorelaxation and cardiac function regulation [50]. Findings from studies continue to provide additional support to epidemiologic evidence of an association between ambient SO_2_ concentration and various measures of respiratory morbidity in the general population [51]. Although others findings suggested that endogenous SO_2_ was a novel gasotransmitter in the cardiovascular system and provided a new therapy target for cardiovascular diseases [52].

Inhalation of ozone injures the airway and causes inflammation. The strength of evidence for a causal relationship between short-term and long-term exposure to ozone and CV effects was categorized by experts as “below equipoise” [53],[54]. Elevated ambient ozone concentrations have been epidemiologically associated with nasal airway inflammation [55],[56]. In children, Hwang et al. provided evidence that long-term exposure to O_3_ may have a detrimental effect on the development of lung function [57]. In general population, Pride et al. demonstrated an association of increasing ground-level ozone with an increase in clinic visits for adverse respiratory-related effects in the following day (lag day 1) [58]. Our results showed that an increase of 10 µg/m^3^ of O_3_ could cause at lag1 in children under 5 years an increase of 2% of pneumonia, 3% of sore throat, 4% of earaches and 4% of antibiotic prescriptions.

Emerging epidemiological and experimental studies are now suggesting a relationship between air pollution and COVID-19-related outcomes [20]–[26],[59]–[64]. According to specific mechanisms, several studies suggested that air pollution might play a role in COVID-19-related morbidity and mortality. Findings from studies in Italian regions and provinces suggest that if weather conditions are stable and concentrations of PM are high, the virus could create clusters with PM [62]. Studies from Germany, Spain, UK, France, Italy and China reported a positive association between PM concentrations and the number of SARS-CoV-2 infected cases, while a negative association was found for O_3_ [21]. In a study, covering Italy, Spain, Germany and France, NO_2_ was found to be related to COVID-19 mortality that revealed 83% of COVID-19 fatalities occurred in the regions with the highest NO_2_ levels [63]. Regarding the role of long-term exposure to air pollution, an Italian study reported that mean levels of NO_2_, O_3_, and PM during the past 4 years, were correlated with the number of COVID-19 cases [64].

## 5. Conclusions and limitations

This study highlights that small reduction in the level of risk factors may yield a great benefit for the health of the Great Casablanca population. A reflection on the opportunities to develop feasible actions are needed in order to adopt the more stringent WHO targets. Decision-makers should prioritize the development and implementation of interventions in order to enhance the monitoring of ambient air quality at local level and sensitize and educate citizens to raise awareness about the impact of air pollution on health. Improving air quality would improve peoples' health quality of life, reduce absenteeism, increase the work productivity and reduce the public health problems.

However, there are several limitations to this study that may influence the interpretation of the results. Regarding the ambient exposure of the population, our results are affected by the ecological fallacy because of using ecological data on exposure to pollutants. Equally, assuming independence of the effects of pollutants could overestimate our results. One of the strengths of this study is its inclusion of a large and socio-demographically diverse population, which minimizes the likelihood of selection bias and may enhance the generality. Also, there is need for more detailed studies to evaluate the relationship between air pollution, atmospheric stability/turbulence and the spread of COVID-19 in Casablanca.
